# HBV-induced miR-4461 downregulation correlates with elevated fibrinogen alpha chain expression in hepatocellular carcinoma

**DOI:** 10.1007/s00535-026-02433-1

**Published:** 2026-04-30

**Authors:** Masatake Kanai, Aiko Sakai, Tomoko Date, Yoshihiko Aoki, Fuminori Mihara, Takashi Kokudo, Fuyuki Inagaki, Nobuyuki Takemura, Norihiro Kokudo, Masaya Sugiyama

**Affiliations:** 1 Department of Viral Pathogenesis and Controls, National Institute of Global Health and Medicine, Japan Institute for Health Security, Tokyo, 162-8655 Japan; 2 Faculty of Life Sciences, Tokyo University of Agriculture, Tokyo, Japan; 3 Department of Gastroenterology and Hepatology, National Kohnodai Medical Center, Japan Institute for Health Security, Chiba, Japan; 4 Department of Surgery, National Center for Global Health and Medicine, Japan Institute for Health Security, Tokyo, Japan; 5 Department of Hepato-Biliary-Pancreatic Surgery and Pediatric Surgery, Saitama Medical Center, Saitama Medical University, Saitama, Japan

**Keywords:** MiR-4461, Hepatitis B virus, Hepatocellular carcinomas, Fibrinogen alpha chain

## Abstract

**Background:**

Hepatitis B virus (HBV) infection remains a leading cause of hepatocellular carcinoma (HCC), yet the molecular mechanisms underlying HBV-mediated hepatocarcinogenesis are not fully understood. This study focused on miR-4461, which is encoded within the intronic region of PCBD2 gene, and investigated its role in HBV-derived HCC.

**Methods:**

miR-4461 expression was examined in HBV-expressing hepatoma cell lines and in HBV-infected primary human hepatocytes. Functional analysis evaluated the effects of miR-4461 overexpression or inhibition on hepatocyte proliferation. Candidate targets were screened, and fibrinogen alpha chain (FGA) was tested for regulation by miR-4461. Circulating miR-4461 and plasma FGA were measured in patients with chronic viral hepatitis, including those with HBV-related HCC; in the HBV-HCC cohort, FGA was also assessed before and after surgical tumor resection.

**Results:**

miR-4461 expression was significantly reduced in HBV-expressing hepatoma cells and HBV-infected primary hepatocytes. Overexpression of miR-4461 inhibited hepatocyte proliferation, whereas its inhibition enhanced proliferation, indicating a tumor-suppressive function. FGA was identified as a downstream target; in hepatoma cells, FGA protein levels were regulated by miR-4461. Clinically, circulating miR-4461 levels were significantly lower in HBV-infected individuals, particularly in those with HBV-related HCC. In contrast, plasma FGA protein levels were markedly elevated in HBV-related HCC and decreased significantly after tumor resection.

**Conclusions:**

These findings suggest a novel regulatory axis in HBV-associated HCC involving HBV-induced suppression of miR-4461 and subsequent upregulation of FGA. Both miR-4461 and FGA were associated with disease status, supporting their potential utility as biomarkers or therapeutic targets in HBV-derived HCC.

**Supplementary Information:**

The online version contains supplementary material available at 10.1007/s00535-026-02433-1.

## Introduction

Hepatitis B virus (HBV) infection remains a major global health burden, affecting approximately 254 million individuals worldwide and causing over 800,000 deaths annually, primarily due to complications, such as cirrhosis and hepatocellular carcinoma (HCC) [1]. HBV is a hepatotropic DNA virus that establishes chronic infection in hepatocytes. The oncogenic potential of HBV is attributed to multiple mechanisms, including chronic necro-inflammatory injury, immune-mediated hepatocyte turnover [2], and integration of viral DNA into the host genome [3], which can disrupt tumor suppressor genes and activate oncogenes [4].

MicroRNAs (miRNAs) are small, noncoding RNAs that play critical roles in post-transcriptional gene regulation by targeting messenger RNAs (mRNAs) for degradation or translational repression [5, 6]. As a result, miRNAs have attracted significant attention as potential biomarkers for a wide range of diseases, including cancer, cardiovascular disorders, and viral infections [7, 8]. Their remarkable stability in body fluids such as blood, urine, and saliva, along with their tissue- and disease-specific expression profiles, makes them ideal candidates for non-invasive diagnostic and prognostic applications. In liver diseases, including HBV infection and HCC, miRNAs are recognized as important modulators of inflammation, fibrosis, cell proliferation, and oncogenic transformation [9–11]. Distinct miRNA expression profiles have been identified in HBV-related HCC, with several tumor-suppressive miRNAs being downregulated and oncogenic miRNAs being upregulated [12–14]. Among the less-studied miRNAs, miR-4461 has recently emerged as a context-dependent regulator in cancer biology. While miR-4461 has been reported to promote tumor progression in ovarian cancer by targeting PTEN [15], it appears to act as a tumor suppressor in other cancers such as renal cell carcinoma and gallbladder cancer [16], where it inhibits cell proliferation, migration, and self-renewal by targeting genes like *PPP1R3C* and *EGFR* [17]. In HCC, miR-4461 has been shown to be downregulated, particularly in liver cancer stem cell populations, and suppress tumorigenesis by directly targeting SIRT1 [18]. However, its role in the context of HBV infection and HBV-associated hepatocarcinogenesis remains unclear.

Despite increasing recognition that HBV exerts direct effects on hepatocytes, the microRNA-mediated regulatory programs that may connect viral persistence to hepatocellular transformation remain poorly defined. We therefore investigated whether HBV alters hepatocyte microRNA expression in a biologically meaningful manner, with particular focus on miR-4461. By interrogating its downstream regulatory network and clinical relevance, we aimed to uncover a previously unrecognized molecular axis potentially involved in HBV-associated hepatocarcinogenesis.

## Materials and methods

### Plasmid construction

The HBV genotype C expression vector pUC19/C_JPNAT, which contains a replication-competent 1.24-fold overlength HBV genome, was used for in vitro HBV expression experiments. This plasmid was originally constructed and described in a previous study [19]. The vector was propagated in *Escherichia coli* DH5α and purified using a plasmid maxi prep kit (Qiagen, Hilden, Germany) according to the protocol from the manufacturer.

To generate plasmids that express only a single gene of HBV, we introduced stop codons into specific open reading frames of the replication-competent construct pUC19/C_JPNAT. For the construct expressing only the PreC/C gene, expressions of PreS/S, POL, and X genes were disrupted by introducing stop codons into the respective coding regions [20–23]. Similarly, constructs expressing only PreS/S, POL, or X were generated by selectively preserving the target gene while introducing stop codons into the remaining HBV genes to prevent their expression.

### Cell culture and reagents

HuH7 and HepG2 cells were cultured in Dulbecco’s modified Eagle’s medium (DMEM) (Sigma, St. Louis, MO, USA) supplemented with 10% fetal bovine serum and 1% (v/v) penicillin-streptomycin at 37 °C in a humid atmosphere of 5% CO_2_. PHHs (PhenixBio, Higashi-Hiroshima, Japan) were grown in DMEM supplemented with 2% fetal bovine serum, 20 mM HEPES, 44 mM NaHCO_3_, 15 μg/mL L-proline, 0.25 μg/mL insulin, 50 nM dexamethasone, 5 ng/mL EGF, 0.1 mM Asc-2P and 2% DMSO (2% DMSO-supplemented hepatocyte clonal growth medium (dHCGM)) at 37 °C and 5% CO_2_ [24].

### Cell transfection

HuH7 and HepG2 cells were transfected with plasmids of HBV genotype C or a negative control plasmid pUC19 using Fugene6 transfection reagents (Promega, Madison, WI) according to the protocol from the manufacturer [19, 25]. Transfection efficiency was measured by co-transfection with a reporter plasmid expressing secreted alkaline phosphatase (SEAP). SEAP activity was then measured in the culture supernatant and normalized with subsequent SEAP measurements from culture supernatant using a SEAP reporter assay kit (TOYOBO, Osaka, Japan). The range of SEAP activity values was within 10%. If the value of even one sample was over 10%, the experiment was omitted to match the background condition of the experiment. All data are based on the results of at least three independent experiments.

A double-stranded hsa-miR-4461 mimic (miRBase accession MIMAT0018983: GAUUGAGACUAGUAGGGCUAGGC), a single-stranded chemically modified antisense oligonucleotide targeting miR-4461 (AS-miR-4461; GCCTAGCCCTACTAGTCTCAATC), and the respective scrambled negative controls (UCGUUAAUCGGCUAUAAUACGC) (Integrated DNA Technologies, Hokkaido, Japan) were used in this study. Lipofectamine RNAiMAX (Thermo Fisher Scientific, Waltham, MA) was used in accordance with the instructions from the manufacturer. To monitor transfection efficiency, a fluorescein-labeled non-targeting control oligonucleotide (Integrated DNA Technologies) was transfected in parallel under identical conditions and visualized 6 h post-transfection by fluorescence microscopy; experiments with < 80% FAM-positive cells were excluded from analysis. All experiments were conducted independently at least three times and data are presented as mean ± standard deviation (SD).

### HBV infection

HBV particles (genotype C) obtained from the serum of a chimeric mouse with human hepatocytes were prepared for infection assay [19, 25]. PHHs were cultured on type I collagen-coated 24-well plates. PHHs were incubated with dHCGM containing the inoculum in the presence or absence of 4% polyethylene glycol 8000 (Promega, Tokyo, Japan) [26]. At day 1, PHHs were infected with inoculum containing 1 ×10^5^ copies of HBV. At 2 days post-infection, PHHs were washed with DMEM, then fresh dHCGM was added. At 4 days post-infection, PHHs were harvested to obtain total RNA using ISOGEN reagents (FUJIFILM, Tokyo, Japan).

### Cell count assay

The Cell Counting Kit-8 (CCK-8) assay (FUJIFILM) was performed to assess cell proliferation according to the instructions from the manufacturer. HuH7 and HepG2 cells were seeded in a 96-well culture plate. At 48 h post-miRNA transfection, CCK-8 solution was added to each well. After 2 h of incubation at 37 °C, absorbance was measured at 450 nm with a microplate reader (Molecular Devices, San Jose, CA). CCK-8 assay results were expressed as optical density values, reflecting the number of viable cells.

### RNA real-time quantitative PCR (RT-qPCR)

Total RNA was extracted from cells using ISOGEN according to the protocol defined by the manufacturer. RNase-Free DNase (Promega, Madison, WI) was used to remove remaining genomic DNA from the isolated total RNA. RT-qPCR analysis of miR-4461 expression was carried out using commercial TaqMan MicroRNA assay kits (Thermo Fisher Scientific) according to the instructions from the manufacturer. Results were normalized to U6 snRNA using the comparative threshold cycle (Ct) method.

For mRNA quantification, total RNA was converted to cDNA using SuperScript IV Reverse Transcriptase (Thermo Fisher Scientific) according to the protocol provided by the manufacturer. *PCBD2* mRNA levels were quantified with pre-designed TaqMan Gene Expression Assays (Thermo Fisher Scientific) and TaqMan Universal PCR Master Mix II. Amplification was carried out on a LightCycler480 System (Roche, Basel, Switzerland). Relative expression was calculated by the comparative Ct (2^-ΔΔCt) method using GAPDH as the endogenous control.

Each sample was analyzed in technical duplicate, and the data represent the mean ± SD from at least three independent biological experiments.

### Immunoblotting

Cells were washed twice with ice-cold PBS and lysed in RIPA buffer (FUJIFILM) supplemented with a protease- and phosphatase-inhibitor cocktail (Roche). Protein concentrations were determined with the Pierce BCA Protein Assay Kit (Thermo Fisher Scientific). Protein extracts were separated in sodium dodecyl sulfate–polyacrylamide gel electrophoresis, transferred to polyvinylidene difluoride membranes, and incubated with the primary antibodies to FGA (ab92572; Abcam, Cambridge, MA), Cytochrome P450 2C8 (CYP2C8) (ab88904; Abcam), Lipopolysaccharide-Binding Protein (LBP) (ab169776; Abcam), and GAPDH (ab8245; Abcam) as a loading control. Band intensities were quantified using ImageJ software (NIH, Bethesda, DC), with target protein signals normalized to GAPDH and expressed relative to control samples. Each experiment was performed independently at least three times and data are presented as mean ± SD.

### Luciferase assay

HeLa cells were used to evaluate the interaction between miR-4461 and 3’ UTR of FGA mRNA via a dual-luciferase reporter system. Cells were seeded in 24-well plates at a density of 1 × 10^5^ cells per well and incubated overnight. On the following day, cells were co-transfected with 100 ng of the pmirGLO reporter plasmid containing the firefly luciferase–FGA 3’ UTR fusion construct and 200 ng of either pmR-ZsGreen1/miR-4461 or the pmR-ZsGreen1/miR-NTC as a negative control, using Lipofectamine 3000 (Thermo Fisher Scientific) according to the manufacturer’s instructions. After 24 hours of incubation, cells were lysed in passive lysis buffer (Promega), and luciferase activities were measured using the dual-luciferase reporter assay system (Promega). Firefly luciferase activity was normalized to Renilla luciferase activity, which is constitutively expressed from the same vector, to correct for transfection efficiency and cell viability. Luminescence was detected using microplate luminometer (Molecular Devices, San Jose, CA), and relative luciferase activity was calculated as the ratio of firefly to Renilla signal. All experiments were performed in three independent biological replicates, and the results were expressed as mean ± standard deviation.

### ELISA for FGA

All blood samples were run in triplicate and analyzed in a blinded fashion in triplicate. Concentrations of isoform I of FGA were quantified with a Human FGA ELISA Kit (Bioss, Woburn, MA). Standard curves were generated and used to determine concentrations of FGA in the samples analyzed.

### Patients and their samples

For the clinical analysis of miR-4461 and FGA expression, we recruited patients with chronic viral hepatitis based on serological markers. HBV-infected patients were defined as those positive for hepatitis B surface antigen, while hepatitis C virus (HCV)-infected patients were defined as those positive for anti-HCV antibodies, and were confirmed to have no evidence of current or past HBV infection based on the absence of anti-HBc antibodies. Within each group, patients were further stratified according to the presence or absence of HCC, resulting in four distinct cohorts: HBV with HCC (*n* = 14); HBV without HCC (*n* = 14); HCV with HCC (*n* = 10); and HCV without HCC (*n* = 10). To ensure the integrity of comparisons, patients with HIV co-infection or suspected dual infection with both HBV and HCV were excluded from the study. In the HBV cohort, all patients had received nucleos(t)ide analog therapy and were classified according to the presence or absence of HCC. In the HCV cohort, patients with HCC consisted of individuals who developed HCC during or within three years after antiviral therapy, including interferon-based therapy or direct-acting antivirals (DAAs) such as ledipasvir/sofosbuvir. In contrast, the non-HCC HCV group included patients who had undergone antiviral therapy and remained free of HCC for more than three years after treatment. Samples from patients without adequate follow-up were excluded from the analysis.

Among eligible individuals, blood samples were collected from those who met predefined clinical criteria for sample quality and disease status. For miRNA quantification, total nucleic acids were extracted from plasma using the miRNeasy Serum/Plasma Kit (Qiagen, Hilden, Germany), following the protocol optimized for the recovery of circulating miRNA as described by the manufacturer. Expression levels of miR-4461 were subsequently analyzed by RT-qPCR. All study procedures were reviewed and approved by the institutional ethics committee of the Japanese Institute for Hepatic Studies (approval nos. NCGM-S-000214 and NCGM-S-000290).

### Statistical analysis

Data are presented as the mean ± SD of at least three independent experiments. Statistical analysis was performed using SPSS 20 and GraphPad Prism 10 software. The results were analyzed using Student's unpaired two-tailed t-test or one-way ANOVA. The values of P < 0.05 were regarded as statistically significant.

## Results

### Downregulation of miR-4461 in HBV-infected cells in vitro

We first investigated RNA expression profile in PHHs infected with HBV. RNA-seq analysis was performed on primary cultured liver cells infected with HBV. Comparative analysis was then conducted based on the presence or the absence of HBV infection (Supplemental Fig. 1A). A substantial change in miR-4461 expression was observed in HBV-infected hepatocytes compared to HBV-negative controls (data not shown). As shown in Supplemental Figure 1B, miR-4461 is located in the intron region of the *PCBD2* gene.

**Fig. 1:**
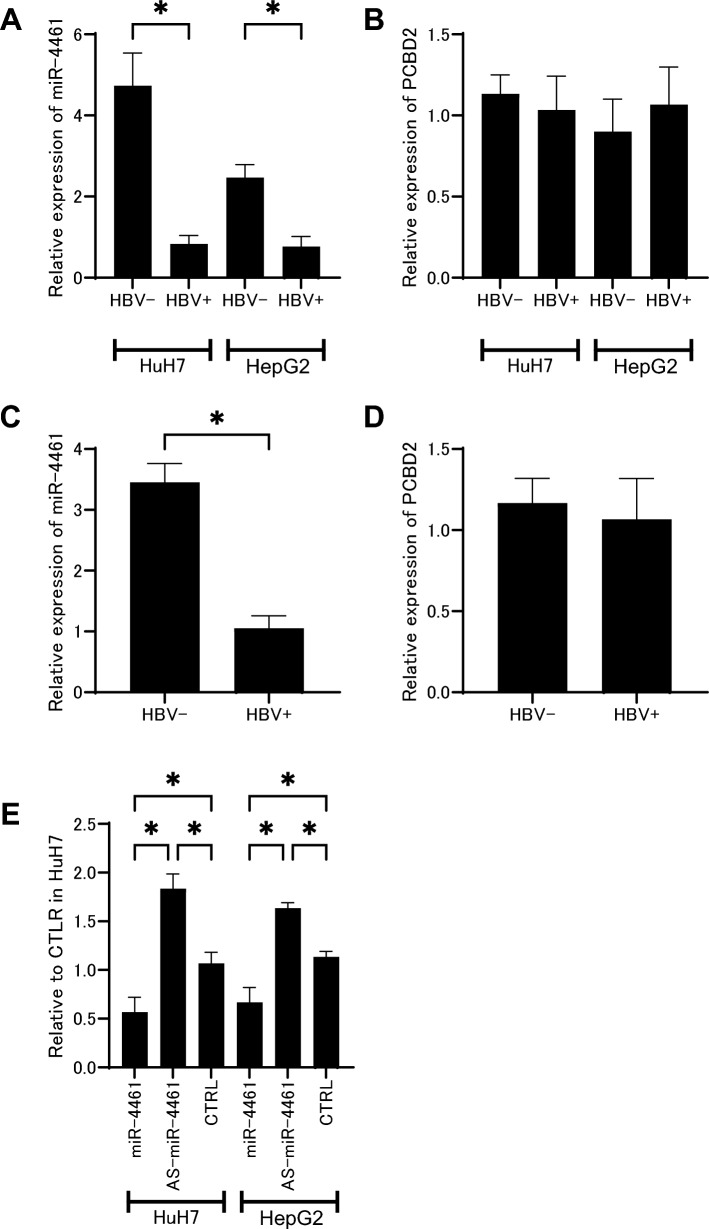
HBV suppresses miR-4461 expression and modulates cell proliferation. **A**, **B** HuH7 and HepG2 cells were transfected with an HBV replication construct (HBV+) or an empty vector control (HBV-). Total RNA was extracted, and expression levels of miR-4461 and PCBD2 were quantified by RT-qPCR. Data are presented as relative expression levels normalized to the HBV+ condition. **C**, **D** Primary human hepatocytes were analyzed for intracellular miR-4461 and PCBD2 mRNA levels by real-time quantitative PCR following infection with or without HBV. **E** Cell proliferation was assessed in HuH7 and HepG2 cells after transfection with miR-4461 mimics or antisense oligonucleotides. Relative proliferation was calculated with untreated cells (CTRL) as the reference. All experiments were performed independently at least three times, and data are shown as mean ± SD. *P < 0.05.

We first confirmed changes in miR-4461 expression in hepatoma cell lines following HBV expression. HuH7 and HepG2 cells were transfected with an HBV-expressing plasmid, and miR-4461 levels were quantified (Fig. 1A). In both cell lines, miR-4461 expression was significantly reduced upon HBV expression. However, expression levels of the *PCBD2* gene, within the intronic region of which miR-4461 is located, remained unchanged across all samples (Fig. 1B). To further validate this observation in a physiologically relevant model, we examined miR-4461 expression in PHHs (Fig. 1C, D). Following infection with HBV particles, intracellular levels of miR-4461 were measured. Consistent with the findings from hepatoma cell lines, miR-4461 expression was also decreased in HBV-infected PHHs. These results indicate that HBV infection or replication suppresses miR-4461 expression in hepatocytes independently of *PCBD2* gene expression. We next investigated the functional role of miR-4461 in cultured cells. Transfection of miR-4461 in HuH7 and HepG2 cells led to significant reductions in cell proliferation compared to control cells (Fig. 1E). Conversely, transfection with antisense oligonucleotides targeting miR-4461 resulted in enhanced cell proliferation relative to controls.

Collectively, these findings suggest that miR-4461 may play a role in regulating hepatocyte growth and maintaining normal cellular properties, and that its downregulation by HBV may contribute to the disruption of cellular homeostasis.

### HBx specifically suppressed miR-4461 expression among HBV gene products

**Fig. 2:**
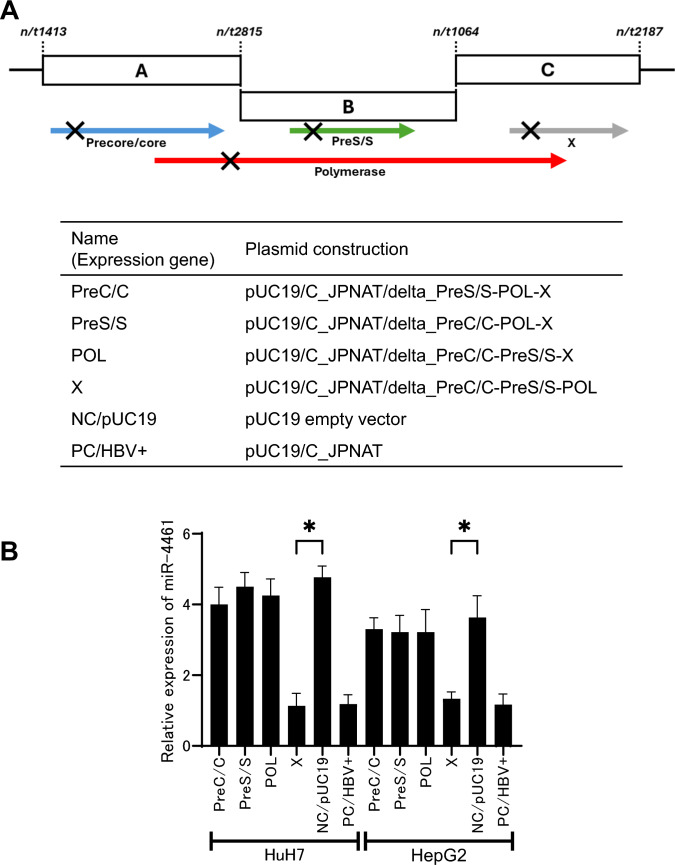
Effect of individual HBV gene expression on miR-4461 levels. **A** Stop codons were introduced into an HBV replication construct (pUC19/C_JPNAT) to generate plasmids expressing only a single HBV gene. The table summarizes each construct name and the corresponding HBV genes disrupted by stop codons. NTC represents the empty pUC19 vector, and PC denotes the full-length HBV expression construct. **B** HuH7 and HepG2 cells were transfected with each construct. Total RNA was extracted from the cells, and miR-4461 expression was quantified by real-time quantitative PCR. Relative expression levels are shown using PC as the reference. Data represent the mean ± SD from three independent experiments. *P < 0.05.

To evaluate which HBV gene was responsible for the suppression of miR-4461, we transfected HuH7 and HepG2 cells with plasmids expressing individual HBV genes (PreC/C, PreS/S, POL, or X) derived from the replication-competent construct pUC19/C_JPNAT. Each construct was engineered to express only one HBV gene by introducing premature stop codons into the other open reading frames as illustrated in Figure 2A. The specific design and corresponding names of each construct are summarized in the table shown in Figure 2A. Among the constructs tested, only expression of the X gene led to a significant reduction in miR-4461 levels in both HuH7 and HepG2 cells, whereas expressions of PreC/C, PreS/S, or POL had no such effect. These results suggest that the HBx protein plays a specific role in mediating the suppression of miR-4461.

### Inhibition of miR-4461 increased FGA expression

**Fig. 3:**
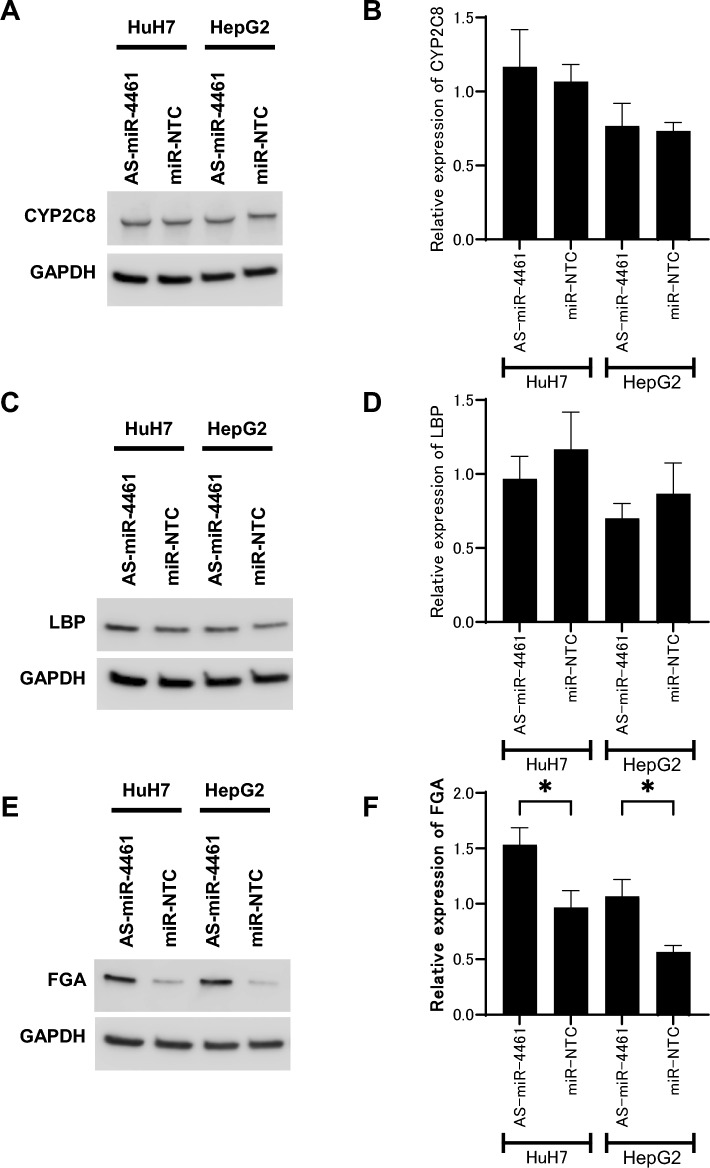
Suppression of miR-4461 by antisense oligonucleotides upregulates FGA expression in hepatocytes. Candidate target genes of miR-4461 were selected based on miRNA-binding prediction databases and predominant expressions in hepatocytes. The regulatory effects of miR-4461 were evaluated in HuH7 and HepG2 cells. Cells were transfected with either an antisense oligonucleotide targeting miR-4461 or a control oligonucleotide. **A**, **B** CYP2C8; **C**, **D** LBP; **E**, **F** FGA. **A**, **C**, **E** Immunoblot analysis of protein expressions. B, D, F) Quantification of immunoblot band intensities, presented as relative expression levels. Data represent the mean ± SD from three independent experiments. *P < 0.05.

Human genes that might be regulated by miR-4461 were searched for through the TargetScan [27], miRDB [28], and Tarbase [29] databases. We compared the predicted miR-4461 target gene lists obtained from three independent databases. Among the top-ranked candidate genes, we prioritized those with liver-enriched or liver-specific expression profiles. Through this selection process, we identified CYP2C8, FGA, and LBP as relevant targets for further validation. To assess whether miR-4461 functionally regulates expression of the predicted target genes, we modulated miR-4461 levels in HuH7 and HepG2 cells and evaluated the corresponding protein expression levels. To inhibit endogenous miR-4461, we transfected cells with an antisense oligonucleotide specifically targeting miR-4461. Immunoblot analysis revealed that protein levels of CYP2C8 and LBP remained unchanged in both HuH7 and HepG2 cells following miR-4461 inhibition (Fig. 3A-D). In contrast, FGA protein levels were notably increased in cells treated with the miR-4461 antisense oligonucleotide compared to control cells (Fig. 3E, F). These results suggest that miR-4461 may selectively suppress FGA expression at the post-transcriptional level, whereas its effects on CYP2C8 and LBP protein expressions may be minimal or regulated through other mechanisms.

### FGA, but not CYP2C8 or LBP, was downregulated by miR-4461

**Fig. 4:**
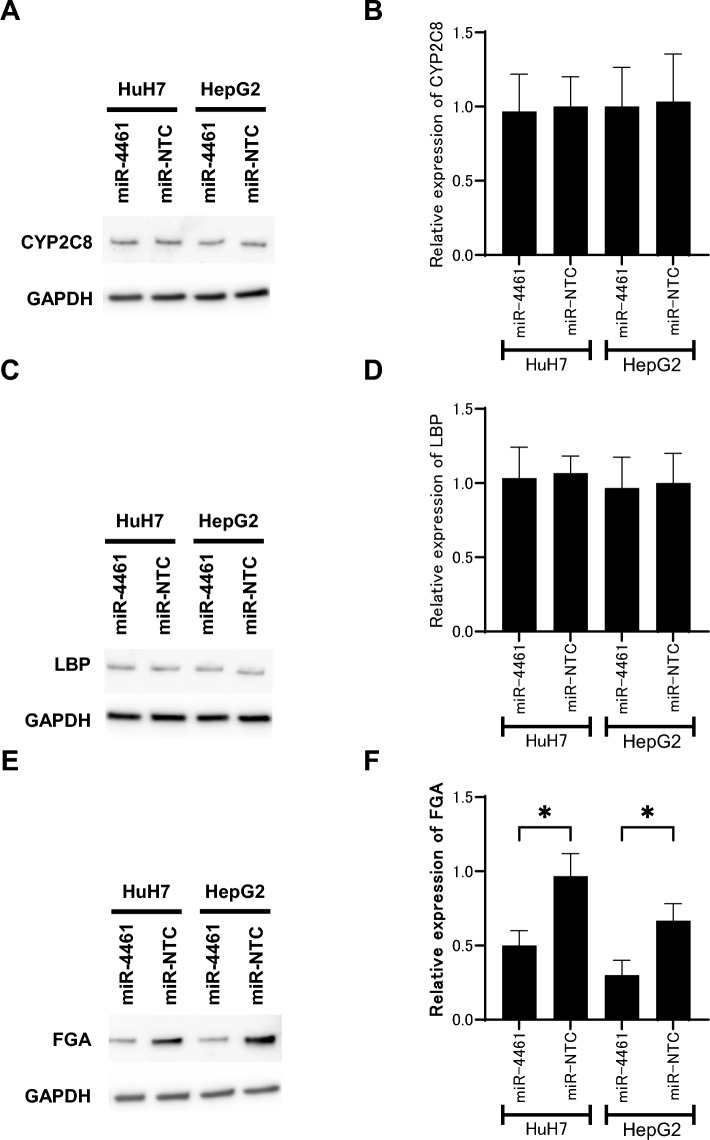
miR-4461 suppressed FGA expression in hepatocytes. Effects of miR-4461 on candidate target genes were evaluated in HuH7 and HepG2 cells. Cells were transfected with either a miR-4461 mimic or a non-targeting control (miR-NTC), and protein expression was assessed. **A**, **B** CYP2C8; **C**, **D** LBP; **E**, **F** FGA. **A**, **C**, **E** Immunoblot analysis of protein expression. B, D, F) Quantification of immunoblot band intensities, expressed as relative values normalized to control. Data are presented as mean ± SD from three independent experiments. *P < 0.05.

To further validate the regulatory effects of miR-4461, we examined changes in target protein expression following miR-4461 transfection in HuH7 and HepG2 cells. Cells were transfected with a miR-4461 or miR-NTC, and protein levels of the candidate targets were assessed by immunoblotting. Consistent with the results from the inhibition experiments, CYP2C8 and LBP protein levels remained unchanged upon miR-4461 overexpression (Fig. 4A-D). In contrast, FGA protein expression was significantly reduced in cells transfected with miR-4461 compared to control cells (Fig. 4E, F). These findings support the conclusion that miR-4461 negatively regulates FGA expression, but has minimal or no impact on CYP2C8 or LBP protein levels in these cell lines.

### Luciferase reporter assay validating miR-4461 targeting of the FGA 3′-UTR

**Fig. 5:**
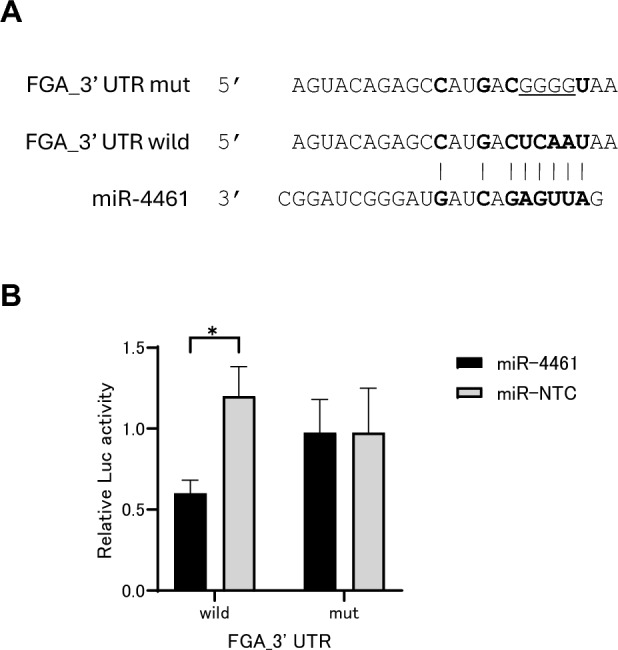
miR-4461 directly targets the FGA 3’-UTR in luciferase assays. **A** Schematic of FGA 3’-UTR sequences. The full-length human FGA 3’-UTR (EBI/Ensembl ENST00000403106.8_7) was cloned downstream of the firefly luciferase gene in a pmirGLO-based vector. A mutant (mut) reporter was generated by introducing point substitutions within the predicted miR-4461 seed-matching sites. **B** Hela cells were co-transfected with the wild-type (wild) or mutant (mut) FGA-3’-UTR reporter and either a miR-4461 expression plasmid or a non-targeting control (miR-NTC). At 24 h, firefly and Renilla activities were measured; firefly signals were normalized to Renilla (internal control) and expressed relative to the miR-NTC condition for each reporter. Data are mean ± SD from 3 independent experiments. *P < 0.05.

The full 3’-UTR of human FGA mRNA was retrieved from the EBI/Ensembl database (ENST00000403106.8_7) and cloned downstream of the firefly luciferase gene in a pmirGLO-based vector. Mutant reporter constructs were generated by introducing point substitutions within the predicted miR-4461 seed-matching sites (Fig. 5A). Hela cells were co-transfected with the wild-type (wild) or mutant (mut) FGA-3’-UTR reporter together with either a miR-4461 expression plasmid or a non-targeting control (miR-NTC). Firefly and Renilla luciferase activities were measured 24 h post-transfection, and firefly signals were normalized to Renilla to control for transfection efficiency. In both cell lines, miR-4461 significantly reduced normalized luciferase activity from the wild FGA-3’-UTR reporter compared with miR-NTC, whereas this repression was abolished in the mut reporter (Figure 5B). These results indicate that miR-4461 directly targets the FGA 3’-UTR at the predicted binding sites.

### Expression of miR-4461 was specifically reduced in HBV-infected and HBV-derived HCC patients

**Fig. 6:**
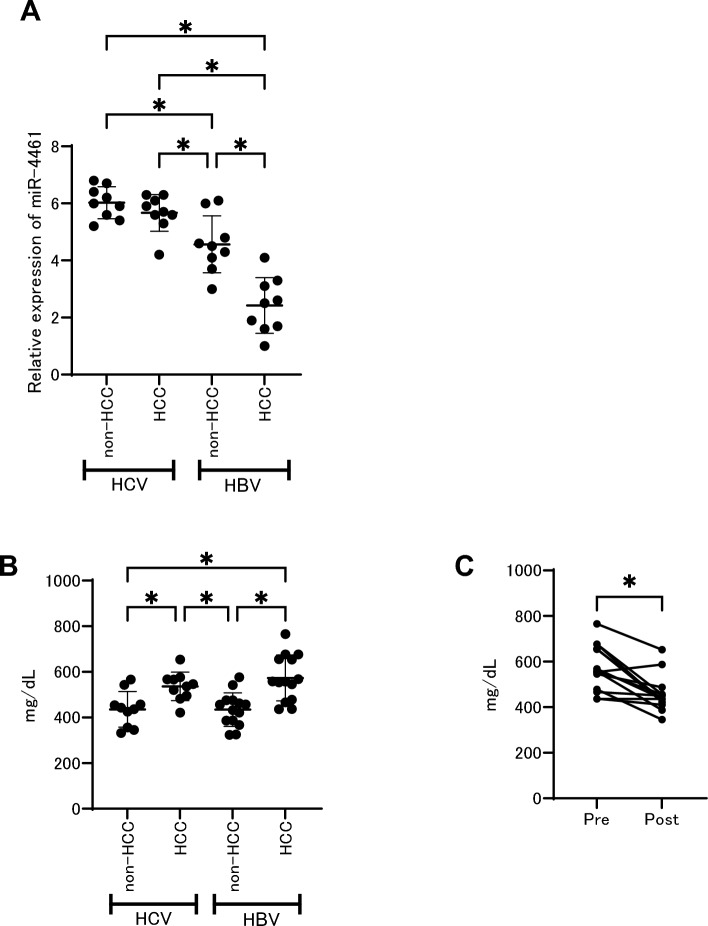
Circulating levels of miR-4461 and FGA in patients with viral hepatitis and HBV-related HCC. **A** Quantification of circulating miR-4461 levels in plasma samples from patients with chronic HBV or HCV infection, with or without HCC (n = 9 per group). Plasma was isolated from whole blood, and total nucleic acids including miRNA were extracted. Levels of miR-4461 were measured using quantitative RT-PCR. **B** Plasma FGA concentrations were quantified by ELISA in the same four patient cohorts (HCV without HCC, HCV with HCC, HBV without HCC, HBV with HCC); n = 10 per HCV cohort and n = 14 per HBV cohort. **C** In patients with HBV-related HCC, plasma FGA levels were measured by ELISA before and after surgical tumor resection, using paired samples from the same 14 patients as in panel B (HBV with HCC cohort; n = 14 pairs). *P < 0.05.

To investigate the clinical relevance of miR-4461, we analyzed its expression in peripheral blood samples from patients with chronic viral hepatitis. We recruited individuals infected with either HBV or HCV and stratified them based on the presence or absence of HCC. Among the recruited patients, we performed quantitative analysis of circulating miR-4461 only in those with archived plasma samples; plasma specimens were available for nine patients in each group. Expression levels of miR-4461 in plasma were quantified and compared across four groups: HCV without HCC, HCV with HCC, HBV without HCC, and HBV with HCC (Fig. 6A). In HCV-infected individuals, miR-4461 expression levels did not differ significantly between those with and without HCC. In contrast, HBV-infected individuals exhibited significantly lower miR-4461 expression compared to HCV-infected individuals, regardless of HCC status. Notably, patients with HBV-related HCC showed the lowest levels of miR-4461 expression among all groups, with significantly reduced levels compared to both HCV-related HCC patients and HBV-infected patients without HCC. These findings suggest that the suppression of miR-4461 is specific to HBV infection and is further exacerbated in the context of HBV-related HCC, highlighting its potential as a biomarker for HBV-derived HCC.

### FGA was highly expressed in HBV-related HCC and decreased after tumor resection

To further assess the clinical relevance of FGA, as a predicted target of miR-4461, we measured its concentration in the blood of patients with chronic viral hepatitis. Plasma FGA levels were compared between patients with HBV- or HCV-related HCC. FGA levels were found to be significantly elevated in patients with HBV-related HCC compared to those with HCV-related HCC (Fig. 6B). To investigate the relationship between tumor burden and FGA levels, we next analyzed plasma samples from HBV-related HCC patients before and after surgical resection of the tumor. A significant decrease in plasma FGA concentration was observed following resection (Fig. 6C), suggesting that the tumor itself may represent a major source of circulating FGA in these patients. This pattern further supports the notion that FGA may be highly expressed in HBV-derived tumor tissue, and elevated blood concentrations may reflect tumor presence. Collectively, these findings imply that FGA may serve as a tumor-associated biomarker in HBV-related HCC, with both tissue-level overexpression and corresponding increased blood levels that decline after tumor removal.

We additionally examined whether circulating miR-4461 expression or plasma FGA concentration was associated with clinical background factors, including inflammation grade (A stage), liver fibrosis stage (F stage), age, and sex, in the HBV and HCV cohorts. No clear associations were observed between these variables and either miR-4461 or FGA levels in either viral etiology (Supplementary Figs. 2 and 3).

## Discussion

In this study, we demonstrated that miR-4461 is consistently downregulated in HBV-infected hepatocytes and in the peripheral blood of patients with HBV-related liver disease, particularly those with HCC. Functional assays revealed that miR-4461 suppresses hepatocyte proliferation, suggesting a tumor-suppressive role. We also identified FGA as a selective, functionally relevant target of miR-4461, showing elevated expression in HBV-related HCC.

Previous studies have demonstrated tumor-suppressive functions of miR-4461 in various cancer types. In ovarian cancer, miR-4461 was shown to inhibit proliferation, metastasis, and cisplatin resistance by targeting PTEN [15]. Similarly, miR-4461 suppressed tumor progression through the EGFR/AKT signaling pathway in gallbladder carcinoma [16], and inhibited tumorigenesis via downregulation of PPP1R3C in renal cell carcinoma [17]. In HCC, miR-4461 was reported to reduce cancer stem cell expansion and chemo-resistance through targeting SIRT1, based on spheroid culture models [18]. These findings collectively highlighted the tumor-suppressive potential of miR-4461, but primarily focused on tumor cell-intrinsic properties or undifferentiated cellular states. In contrast, our study is the first to demonstrate that miR-4461 is downregulated specifically in HBV-infected differentiated hepatocytes, suggesting a virus-driven regulatory mechanism. Furthermore, we identified FGA as a functionally relevant downstream target, linking HBV infection to altered hepatocyte homeostasis and tumor-associated gene expression. This HBV–miR-4461–FGA axis uncovers a novel virus–host interaction that may contribute to hepatocarcinogenesis, distinguishing our work from previous reports that did not consider viral etiology or hepatocyte differentiation status.

Among the predicted targets, FGA was validated as a key downstream effector of miR-4461. Its expression correlated inversely with miR-4461 in vitro, and was strongly expressed in HBV-related HCC patients, with plasma levels decreasing after resection. This implicates FGA as a tumor-derived, blood-detectable molecule.

Previous studies have identified FGA as a prognostic marker in several cancers, including colorectal, gastric, liver, and oral squamous cell carcinoma [30–34]. Our findings regarding FGA expression are consistent with previous reports indicating that elevated FGA levels are associated with poor prognosis in several cancers [30, 32–34], except for one report on undifferentiated hepatic cells [31]. A recent study also demonstrated the utility of FGA as a circulating biomarker in HCC [30]. Overall, our results support the view that increased FGA expression reflects tumor burden and may serve as a biomarker in HBV-derived HCC.

Both miR-4461 and FGA protein exhibit potential as biomarkers for HBV-derived HCC, but are molecularly interconnected, with our data suggesting that miR-4461 directly regulates FGA expression. Given this regulatory relationship, simultaneous measurement of both may not be appropriate, essentially representing redundant assessment of the same biological signal. Further, from a practical standpoint, miRNA-based assays are technically demanding due to sensitivity to sample handling and quality control, whereas FGA protein can be measured more easily and reliably in plasma using standardized ELISA protocols. Thus, FGA protein alone may serve as a more feasible and clinically applicable biomarker.

Some limitations to the study must be acknowledged when interpreting the present findings. First, the upstream mechanisms leading to miR-4461 suppression in HBV infection remain unclear and merit further investigation. Second, although miR-4461 is embedded within an intronic region of the *PCBD2* gene, our data indicate that PCBD2 expression levels are not altered upon HBV infection. This suggests that the downregulation of miR-4461 is likely mediated through independent regulatory mechanisms, rather than host gene co-expression. One potential mechanism may involve HBx, a multifunctional viral protein known to modulate host transcription and epigenetic regulation. HBx has been implicated in the repression of host miRNAs via chromatin remodeling and transcription factor modulation [35, 36] although its specific role in miR-4461 suppression remains to be elucidated. The number of clinical samples analyzed was relatively limited, particularly for subgroup comparisons. Although the observed patterns were consistent, further validation in larger cohorts will be necessary to confirm the role of the miR-4461–FGA axis in HBV-associated liver disease and hepatocarcinogenesis.

In conclusion, our study uncovered a previously unrecognized regulatory axis in HBV-derived HCC, in which HBV infection leads to suppression of miR-4461, resulting in upregulation of FGA protein. This HBV–miR-4461–FGA cascade represents a novel molecular pathway connecting viral infection to host gene regulation and tumor progression. These findings not only provide new mechanistic insights into virus–host interactions, but also identify miR-4461 and FGA as promising candidates for biomarkers or therapeutic agents in patients with HBV-associated HCC.

## Supplementary Information

Below is the link to the electronic supplementary material.

Supplementary file1 (PDF 188 KB)

Supplementary file2 (DOCX 16 KB)

Supplementary file3 (DOCX 21 KB)

## Abbreviations

HBV: Hepatitis B virus
HCV: Hepatitis C virus
HCC: Hepatocellular carcinoma
FGA: Fibrinogen alpha chain
PHH: Primary human hepatocyte
SEAP: Secreted alkaline phosphatase
CCK-8: Cell Counting Kit-8
CYP2C8: Cytochrome P450 2C8
LBP: Lipopolysaccharide-Binding Protein
