# Supplementary figures and images for: HBV-induced miR-4461 downregulation correlates with elevated fibrinogen alpha chain expression in hepatocellular carcinoma
Source: J Gastroenterol. 2026 Apr 30;61(8):1146–58. doi: 10.1007/s00535-026-02433-1 (PMC13407565; doi:10.1007/s00535-026-02433-1)

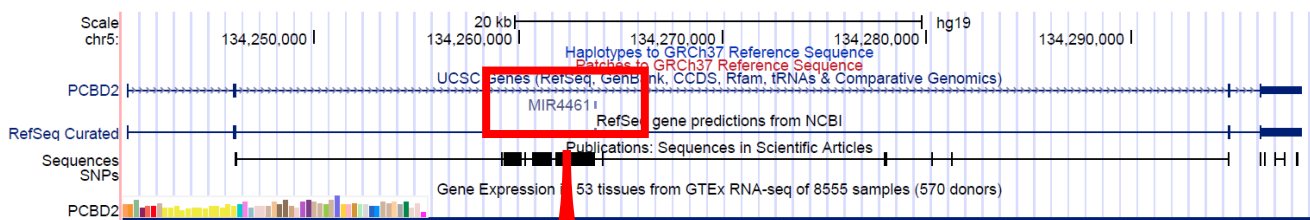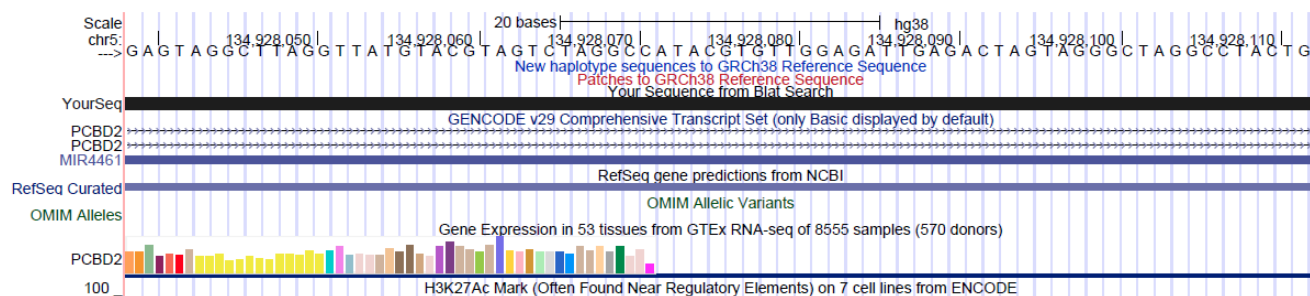

**Supplemental Figure 1**

**A**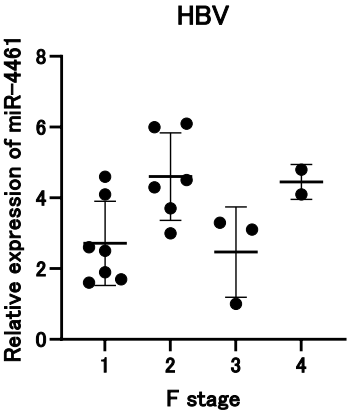**B**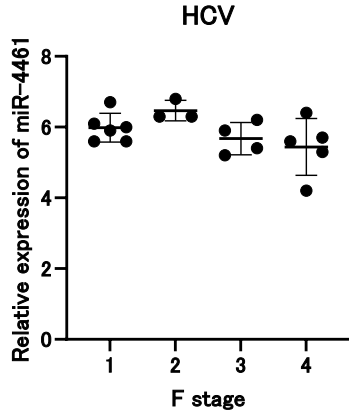**C**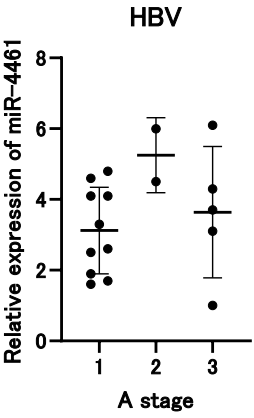**D**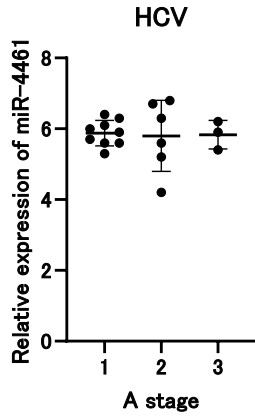**E**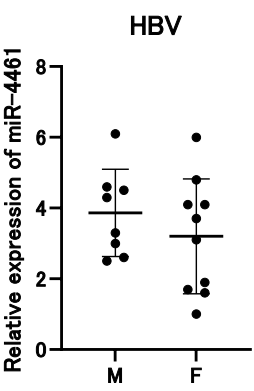**F**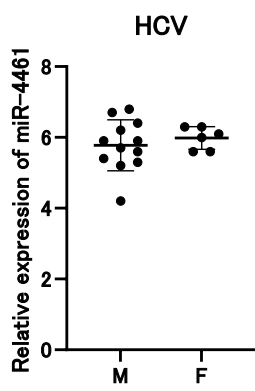**G**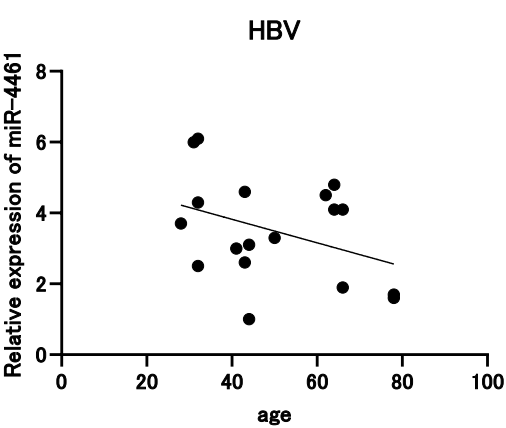**H**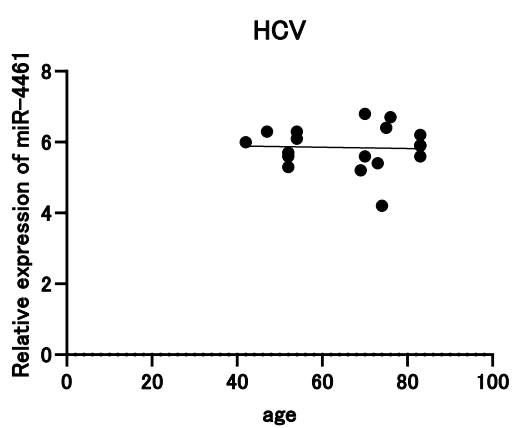

Supplemental Figure 2

**A**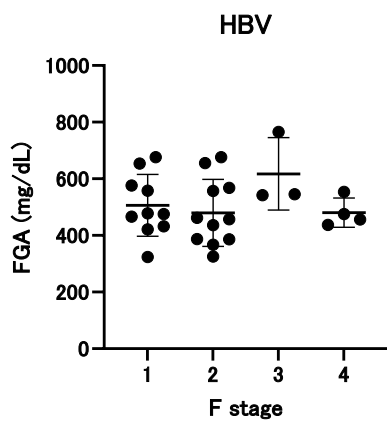**B**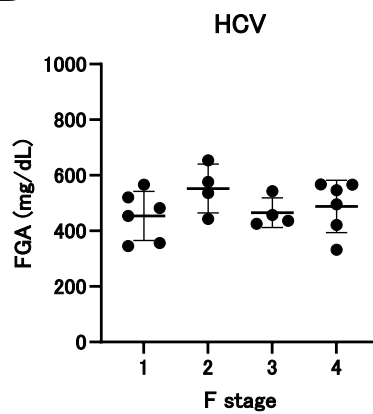**C**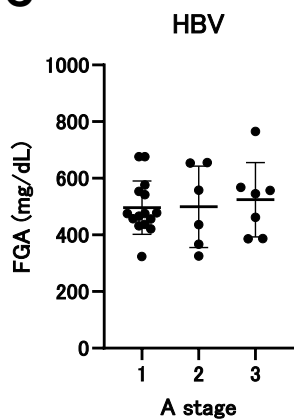**D**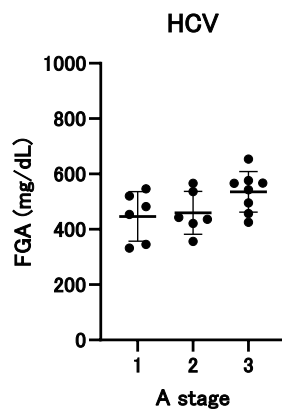**E**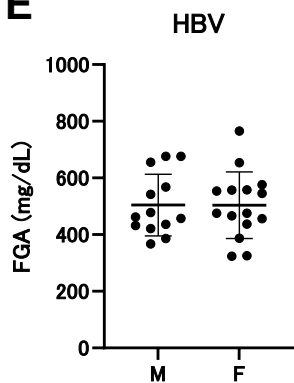**F**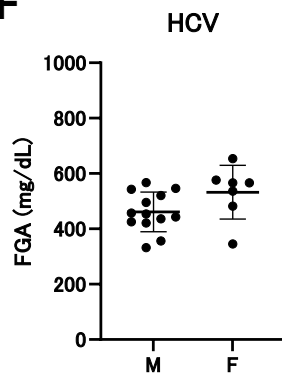**G**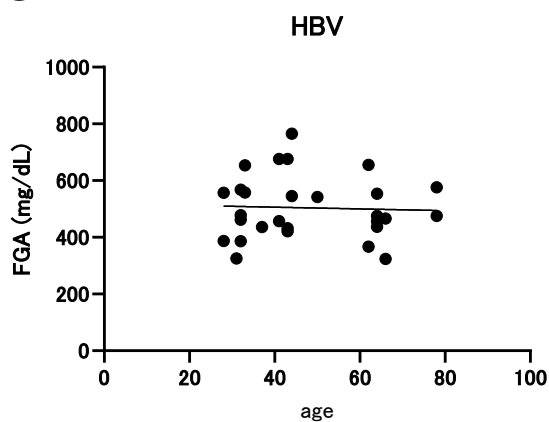**H**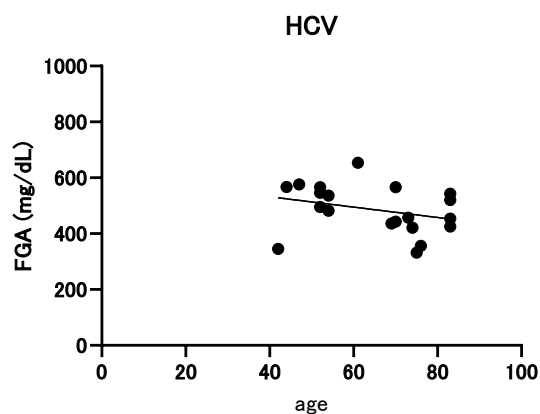

Supplemental Figure 3

Supplement: Supplementary file 1 — Supplementary file1 (PDF 188 KB) [file 535_2026_2433_MOESM1_ESM.pdf]
