# Supplementary material for: HBV-induced miR-4461 downregulation correlates with elevated fibrinogen alpha chain expression in hepatocellular carcinoma
Source: J Gastroenterol. 2026 Apr 30;61(8):1146–58. doi: 10.1007/s00535-026-02433-1 (PMC13407565; doi:10.1007/s00535-026-02433-1)
Supplement: Supplementary file 2 — Supplementary file2 (DOCX 16 KB) [file 535_2026_2433_MOESM2_ESM.docx]

| Name | Sequence |
| --- | --- |
| miR-4461 mimic | GAUUGAGACUAGUAGGGCUAGGC |
| miR-4461 antisense | GCCTAGCCCTACTAGTCTCAATC |
| miR-control | UCGUUAAUCGGCUAUAAUACGC |

# Supplemental Table S1. miRNA sequences used in this study
