# Supplementary material for: HBV-induced miR-4461 downregulation correlates with elevated fibrinogen alpha chain expression in hepatocellular carcinoma
Source: J Gastroenterol. 2026 Apr 30;61(8):1146–58. doi: 10.1007/s00535-026-02433-1 (PMC13407565; doi:10.1007/s00535-026-02433-1)
Supplement: Supplementary file 3 — Supplementary file3 (DOCX 21 KB) [file 535_2026_2433_MOESM3_ESM.docx]

**HBV-induced miR-4461 downregulation correlates with elevated FGA expression in hepatocellular carcinoma**

Masatake Kanai^1, 2^, Aiko Sakai^1^, Tomoko Date^1^, Yoshihiko Aoki^3^, Fuminori Mihara^4^, Takashi Kokudo^4^, Fuyuki Inagaki^4^, Nobuyuki Takemura^4, 5^, Norihiro Kokudo^4^, and Masaya Sugiyama^1^

^1^ Department of Viral Pathogenesis and Controls, National Institute of Global Health and Medicine, Japan Institute for Health Security, Tokyo, Japan

^2^ Faculty of Life Sciences, Tokyo University of Agriculture, Tokyo, Japan

^3^ National Kohnodai Medical Center, Japan Institute for Health Security, Chiba, Japan

^4^ Department of Surgery, National Center for Global Health and Medicine, Japan Institute for Health Security, Tokyo, Japan

^5^ Department of Hepato-Biliary-Pancreatic Surgery and Pediatric Surgery, Saitama Medical Center, Saitama Medical University, Saitama, Japan

**Corresponding author:**

Masaya Sugiyama Ph.D.

Department of Viral Pathogenesis and Controls, National Institute of Global Health and Medicine, Japan Institute for Health Security, Tokyo 162-8655, Japan

Email: [sugiyama.m@jihs.go.jp](mailto:sugiyama.m@jihs.go.jp)

# Supplemental Figure 1. Genomic context of miR-4461

A magnified view of the genomic locus corresponding to the miR-4461 coding region, within the intronic region of the PCBD2 gene.

# Supplemental Figure 2. No association between circulating miR-4461 expression and clinical variables in HBV and HCV cohorts

Circulating miR-4461 expression was analyzed in relation to liver fibrosis stage (F stage), inflammation grade (A stage), sex, and age in patients with HBV and HCV infection. miR-4461 expression according to fibrosis stage (F stage) in HBV (A) and HCV (B) cohorts. miR-4461 expression according to inflammation grade (A stage) in HBV (C) and HCV (D) cohorts. Comparison of miR-4461 expression between male (M) and female (F) patients in HBV (E) and HCV (F) cohorts. Correlation between miR-4461 expression and age in HBV (G) and HCV (H) cohorts.

Each dot represents an individual patient. Data are shown as mean ± SD. Correlations with ordinal or continuous variables were evaluated using Spearman’s rank correlation test, and comparisons by sex were performed using the Mann–Whitney U test. No statistically significant associations were observed between miR-4461 expression and any of the clinical variables. In addition, the coefficients of determination (R²) for the regression analyses in supplemental figure 2G and 2H were < 0.15 and < 0.1, respectively, indicating no meaningful correlation.

# Supplemental Figure 3. No association between plasma FGA concentration and clinical variables in HBV and HCV cohorts

Plasma FGA levels were analyzed in relation to liver fibrosis stage (F stage), inflammation grade (A stage), sex, and age in patients with HBV and HCV infection. Plasma FGA concentration according to fibrosis stage (F stage) in HBV (A) and HCV (B) cohorts. FGA concentration according to inflammation grade (A stage) in HBV (C) and HCV (D) cohorts. Comparison of FGA concentration between male (M) and female (F) patients in HBV (E) and HCV (F) cohorts. Correlation between FGA concentration and age in HBV (G) and HCV (H) cohorts.

Each dot represents an individual patient. Data are shown as mean ± SD. Correlations with ordinal or continuous variables were evaluated using Spearman’s rank correlation test, and comparisons by sex were performed using the Mann–Whitney U test. No statistically significant associations were observed between plasma FGA concentration and any of the clinical variables. In addition, all R² values were < 0.1, indicating no meaningful correlation (G and H).
